# Identification of a novel aminoglycoside *O*-nucleotidyltransferase AadA33 in *Providencia vermicola*

**DOI:** 10.3389/fmicb.2022.990739

**Published:** 2022-09-13

**Authors:** Chunlin Feng, Mengdi Gao, Weiyan Jiang, Weina Shi, Anqi Li, Shuang Liu, Lei Zhang, Xueya Zhang, Qiaoling Li, Hailong Lin, Junwan Lu, Kewei Li, Hailin Zhang, Yunliang Hu, Qiyu Bao, Xi Lin

**Affiliations:** ^1^The Second Affiliated Hospital and Yuying Children’s Hospital, Wenzhou Medical University, Wenzhou, China; ^2^Key Laboratory of Medical Genetics of Zhejiang Province, Key Laboratory of Laboratory Medicine, Ministry of Education, School of Laboratory Medicine and Life Sciences, Wenzhou Medical University, Wenzhou, China; ^3^Medical Molecular Biology Laboratory, School of Medicine, Jinhua Polytechnic, Jinhua, China

**Keywords:** aminoglycoside *O*-nucleotidyltransferase, AadA33, *Providencia vermicola*, enzyme kinetics, novel antimicrobial resistance gene

## Abstract

A novel chromosome-encoded aminoglycoside *O*-nucleotidyltransferase AadA33 was identified in *Providencia vermicola* strain P13. The AadA33 shares the highest amino acid identity of 51.28% with the function characterized AadA31. Antibiotic susceptibility testing and enzyme kinetics analysis revealed that the function of AadA33 is to mediate spectinomycin and streptomycin resistance. The recombinant strain harboring *aadA33* (pUCP20-*aadA33*/*Escherichia coli* DH5α) displayed >256- and 128-fold increases in the minimum inhibitory concentration levels to spectinomycin and streptomycin, respectively, compared with the control strains pUCP20/DH5α. Enzyme kinetic parameters manifested the substrate of AadA33 including spectinomycin and streptomycin, with *k*_cat_/*K*_m_ of 3.28 × 10^4^ (M^−1^ s^−1^) and 3.37 × 10^4^ (M^−1^ s^−1^), respectively. Bioinformatics analysis revealed its structural mechanism of antimicrobial resistance, genetic context, and phylogenetic relationship with other aminoglycoside *O*-nucleotidyltransferases. This study of AadA33 contributed to understanding the function and resistance mechanism of aminoglycoside *O*-nucleotidyltransferase.

## Introduction

Genus *Providencia* is Gram-negative opportunistic pathogens of the family Morganellaceae, which could be isolated from a wide range of organisms and environments. *Providencia vermicola* was first isolated from a nematode *Steinernema thermophilum* in New Delhi, India (Somvanshi et al., 2006). However, unlike the problematic ESKAPEE pathogens (*Enterococcus faecium*, *Staphylococcus aureus*, *Klebsiella pneumoniae*, *Acinetobacter baumannii*, *Pseudomonas aeruginosa*, *Enterobacter* species, and *Escherichia coli*) (Rice, 2008), *Providencia Providencia rettgeri* (Tshisevhe et al., 2016; Shin et al., 2018) and *Providencia stuartii* (Douka et al., 2015; Oikonomou et al., 2016), *P. vermicola* is rarely involved in the nosocomial outbreak (Lupande-Mwenebitu et al., 2021). At the time of writing, there are three genomes of *P. vermicola* deposited in the NCBI database and only one is the complete genome. Aminoglycoside antibiotics are traditional broad-spectrum Gram-negative antibacterial medications that inhibit protein synthesis. Mechanisms of resistance to aminoglycosides mainly include aminoglycoside modifying enzymes (Ramirez and Tolmasky, 2010), increased efflux (Aires et al., 1999), reduced uptake, or decreased permeability (Over et al., 2001), and alterations of 16S rRNA (Doi et al., 2016). At present, the most common mechanism of resistance to aminoglycosides is the inactivation of these antibiotics mediated by various aminoglycoside modifying enzymes (Ramirez and Tolmasky, 2010). Based on the site of modification (Shaw et al., 1993), aminoglycoside modifying enzymes could be classified into aminoglycoside *N*-acetyltransferases (AACs), aminoglycoside *O*-nucleotidyltransferases (ANTs), and aminoglycoside *O*-phosphotransferases (APHs).

ANTs mediate the covalent modification of aminoglycoside antimicrobial hydroxyl group by ATP-dependent transfer of AMP. There are five classes of ANTs, namely ANT(6), ANT(9), ANT(4′), ANT(3″), and ANT(2″), which catalyze nucleotidylylation at the hydroxyl group at positions 6, 9, 4, 3, and 2, respectively. ANT(3″)-Ia (also commonly named AadA) is the most common ANT enzyme of the ANT(3″) family. Up to date, more than 20 types of genes encoding ANT(3″)-Ia enzymes have been identified.

In this study, the function and molecular characteristics of a novel aminoglycoside *O*-nucleotidyltransferase gene (designated *aadA33*) encoded on the chromosome of a *P. vermicola* strain were characterized.

## Materials and methods

### Bacterial strains and plasmids

*Providencia vermicola* P13 was isolated from the blood of an inpatient diagnosed with idiopathic thrombocytopenic purpura at a hospital in Wenzhou, China. Species identification of *P. vermicola* P13 was conducted by the VITEK 2 Compact instrument (bioMerieux, Inc., Craponne, France), 16S rRNA gene sequence, and whole-genome average nucleotide identity (ANI) (Konstantinidis and Tiedje, 2005; Richter and Rosselló-Móra, 2009). The strains and plasmids used in this work are listed in Table 1.

**Table 1:** Bacteria and plasmids used in this work.

Strain and plasmid	Description	Reference
P13	The wild-type strain of *Providencia vermicola* P13	This work
DH5α	*Escherichia coli* DH5α as a host for cloning of the *aadA33* gene	Our laboratory collection
BL21	*Escherichia coli* BL21 as a host for expression of the *aadA33* gene	Our laboratory collection
ATCC 25922	*Escherichia coli* ATCC 25922 as quality control for antimicrobial susceptibility testing	Our laboratory collection
pUCP20-*aadA33*/DH5α	DH5α carrying the recombinant plasmid pUCP20-*aadA33*	This work
pColdI-*aadA33*/BL21	BL21 carrying the recombinant plasmid pColdI-*aadA33*	This work
pUCP20	Cloning vector for the PCR products of the *aadA33* gene with its upstream promoter region, AMP^r^	Our laboratory collection
pColdI	Expression vector for the PCR products of the ORF of the *aadA33* gene, AMP^r^	Our laboratory collection

r, resistance; AMP, ampicillin; ORF, open reading frame.

### Antibiotic susceptibility testing

The minimum inhibitory concentrations (MICs) were determined with the agar dilution method recommended by the Clinical and Laboratory Standards Institute (CLSI) guidelines, and the results were interpreted according to the CLSI M100 (31st Edition, 2021), the European Committee on Antimicrobial Susceptibility Testing (version 11.0, 2021) and National Antimicrobial Resistance Monitoring System for Enteric Bacteria breakpoint criteria.^1^ *E. coli* ATCC 25922 was used as a reference strain for quality control. All tested antimicrobials in this work were listed in Table 2, including one aminocyclitol (spectinomycin); ten aminoglycosides (streptomycin, neomycin, sisomicin, ribostamycin, tobramycin, gentamicin, amikacin, kanamycin, paromomycin and micronomicin); six β-lactams (ampicillin, cefoxitin, cefepime, ceftazidime, meropenem and aztreonam); one quinolone (levofloxacin); one phenicol (chloramphenicol); two tetracyclines (tetracycline and tigecycline); one phosphonic acid (fosfomycin), and one polymyxin (polymyxin E). All the antimicrobials were human drugs bought from a pharmacy or hospital.

**Table 2:** MICs of 23 antimicrobials for 5 strains (μg/ml).

Drug class	Antimicrobial^a^	ATCC 25922	DH5α	pUCP20/DH5α	pUCP20-*aadA33*/DH5α	*P. vermicola* P13
Aminocyclitol	Spectinomycin	8	8	8	>2048	>1,024
Aminoglycoside	Streptomycin	4	2	2	256	512
	Neomycin	1	1	1	1	1,024
	Sisomicin	0.25	0.25	0.25	0.25	32
	Ribostamycin	2	2	2	2	>1,024
	Tobramycin^b^	0.25	0.25	0.25	0.25	128
	Gentamicin^b^	0.25	0.25	0.5	0.25	64
	Amikacin	1	1	1	1	16
	Kanamycin	1	1	1	1	1,024
	Paromomycin	2	2	2	2	>1,024
	Micronomicin	0.25	0.25	0.25	0.25	128
β-Lactam	Ampicillin^b^^,^^c^	4	2	2	/	1,024
	Cefoxitin	4	2	2	/	64
	Cefepime	<0.125	<0.125	0.5	/	32
	Ceftazidime	0.25	<0.125	0.5	/	1,024
	Meropenem	<0.03	<0.03	<0.03	/	16
	Aztreonam	<0.125	<0.125	0.25	/	0.125
Quinolone	Levofloxacin	<0.03	<0.03	<0.03	/	16
Phenicol	Chloramphenicol	4	4	4	/	128
Tetracycline	Tetracycline^b^^,^^c^	2	2	2	/	64
	Tigecycline^b^^,^^c^	0.25	0.5	0.5	/	16
Phosphonic acid derivative	Fosfomycin	2	2	2	/	512
Polymyxin	Polymyxin E^b^^,^^c^	0.5	0.25	0.25	/	> 1,024

a Information of intrinsic resistance in *Providencia* spp. is only available for *P. rettgeri* and *P. stuartii* in CLSI M100 (31st Edition).

b *P. stuartii* is intrinsically resistant to these antimicrobial agents.

c *P. rettgeri* is intrinsically resistant to these antimicrobial agents.

### Cloning of the *aadA33* gene

The *aadA33* gene along with its promoter region was amplified by PCR with the primers listed in Table 3. The PCR product was digested with *Bam*HI and *Hind*III and ligated into the pUCP20 vector with a T4 DNA ligase cloning kit (Takara Bio, Inc., Dalian, China). The recombinant plasmid was transformed into *E. coli* DH5α by the calcium chloride method, and then the transformant was cultured on Luria-Bertani (LB) agar plates supplemented with 100 μg/ml ampicillin. The size and sequence of the cloned insert were verified by restriction enzyme digestion and Sanger sequencing, respectively.

**Table 3:** Primers for cloning the *aadA33* gene.

Primer^a^	Sequence (5′ → 3′)	Restriction endonuclease	Vector	Annealing temperature (°C)	Amplicon size (bp)
pro-*aadA33*-F	ATCCTGAAGAGTCAGAAAACAACGA		pUCP20	55	1,180
pro-*aadA33*-R	ATTACATGTTGTTGCATTGCGCT		pUCP20		1,180
orf-*aadA33*-F	GGATCCCTGGTGCCGCGCGGCAGCATGAATTTTGAACATATAGACAGCA	*Bam*HI + Thrombin	pColdI	55	818
orf-*aadA33*-R	AAGCTTATGCTGACAGAAAGAAAACGAATATCAATGAATTA	*Hind*III	pColdI		818

a Primers starting with “pro” were used to clone the *aadA33* gene and its promoter region; primers starting with “orf” were used to clone the ORF of the *aadA33* gene.

### Expression and purification of the AadA33 enzyme

AadA33 was overexpressed in *E. coli* BL21/pCold I-*aadA33* and purified as described previously (Qing et al., 2004; Shi et al., 2015). The *aadA33* gene was cloned with a thrombin cleavage site into the pCold I vector under the control of the *cspA* promoter using the cold-shock system (Qing et al., 2004). When the OD_600_ of the culture reached 0.6–0.8 at 37°C, induction of protein expression was triggered by the addition of 1 mM isopropyl-β-d-thiogalactoside, and additional incubation was carried out for 20 h at 16°C. Cells were collected by centrifugation (5,000 × *g*, 10 min) at 4°C, resuspended in lysis buffer (20 mM Tris–HCl, 150 mM NaCl, 3 mM β-mercaptoethanol, 0.5% Nonidet-P-40; pH 8.0), and lysed by sonication. After removing cellular debris by centrifugation (12,000 × *g*, 30 min) at 4°C, lysates were incubated with pre-equilibrated nickel-nitrilotriacetic acid (Ni-NTA) agarose resin (Beyotime Biotechnology, Shanghai, China) for 8 h at 4°C under gentle shaking. Then the recombinant protein was purified by standard Ni-NTA affinity chromatography. The His_6_ tag was removed by incubation with thrombin for 24 h at 37°C. The purity of AadA33 protein was validated by SDS-PAGE, and the protein concentration was examined by a BCA protein assay kit (Thermo Fisher Scientific, Rockford, IL, United States).

### Enzyme kinetics

The kinetic assay used to monitor enzyme activity was performed as reported previously (Kim et al., 2006). The AadA33 activity was measured by coupling the enzymatic reaction to the reactions of UDP-glucose pyrophosphorylase, phosphoglucomutase, and glucose-6-phosphate dehydrogenase. The catalytic activity of AadA33 was assayed by monitoring the accumulation of NADPH at 340 nm with a Synergy™ Neo2 Multi-Mode Microplate Reader (BioTek Instruments, Inc., United States). The reaction mixtures contained 50 mM HEPES (pH 7.5), 10 mM MgCl_2_, 0.2 mM UDP-glucose, 0.2 mM glucose 1,6-bisphosphate, 0.2 mM NADP, 0.2 mM dithiothreitol, 2 units/ml UDP-glucose pyrophosphorylase, 20 units/ml phosphoglucomutase, 20 units/ml glucose-6-phosphate dehydrogenase, 1 mM ATP, 3.41 × 10^−8^ mM of AadA33, and variable concentrations of aminoglycoside (1–150 μM) in a total volume of 0.2 ml. Reactions were initiated by the addition of the AadA33.

### Whole genome sequencing and bioinformatic analysis

Genomic DNA of *P. vermicola* P13 was sequenced by the Illumina NovaSeq and PacBio RS II platforms (Shanghai Personal Biotechnology Co., Ltd., Shanghai, China). The Illumina short reads were assembled by SKESA v2.4.0 (Souvorov et al., 2018). The PacBio long reads were assembled by Trycycler v0.5.1 (Wick et al., 2021) and Flye v2.9-b1768 (Kolmogorov et al., 2019). The quality of the draft genome assembly was improved by Pilon by mapping Illumina short reads to the assembly to correct possible misassembled bases (Walker et al., 2014). ANI was computed using FastANI (Jain et al., 2018). Genes were predicted by Prokka v1.14.6 (Seemann, 2014). DIAMOND v2.0.11 (Buchfink et al., 2021) and NCBI non-redundant protein databases were used to annotate deduced proteins. Resistance Gene Identifier v5.2.0^2^ and the comprehensive antibiotic resistance database (CARD, McArthur et al., 2013) were used to identify antimicrobial resistance genes. Multiple sequence alignment, phylogenetic tree construction, and visualization were conducted using MAFFT v7.490 (Katoh and Standley, 2013), IQ-TREE v 2.0.7 (Minh et al., 2020), and ggtree v3.2.0 (Yu et al., 2017), respectively. The conserved domain of AadA33 was discovered by CD-search.^3^ Visualization of genome map and features was generated in GView Server (Petkau et al., 2010). The figure of the genetic environment surrounding the *aadA33* and *aadA33*-like genes was generated by clinker v0.0.24 (Gilchrist and Chooi, 2021). The molecular weight and p*I* value of AadA33 were predicted using ProtParam.^4^ GNU Parallel (Tange, 2021) and Entrez Direct^5^ were used to access resources in the NCBI databases.

## Results

### General features of the *Providencia vermicola* P13 genome

P13 was initially identified as *P. stuartii* by VITEK 2 Compact instrument. The 16S rRNA gene sequence of the isolate P13 has the highest similarity (98.76% coverage and 99.21% identity) with *P. vermicola* OP1 (NR_042415.1). Furthermore, ANI analysis revealed that *P. vermicola* P13 shares the highest ANI (99.16%) with *P. vermicola* P8538 (NZ_CP048796.1). ANI is the proposed genomic gold standard for prokaryotic species classification (Richter and Rosselló-Móra, 2009) and is used at the NCBI to review taxonomy for prokaryotic genomes (Ciufo et al., 2018). Therefore, along with the clinical laboratory result of the VITEK 2 Compact instrument, P13 was finally classified into *P. vermicola* and named *P. vermicola* P13.

**Figure 1:**
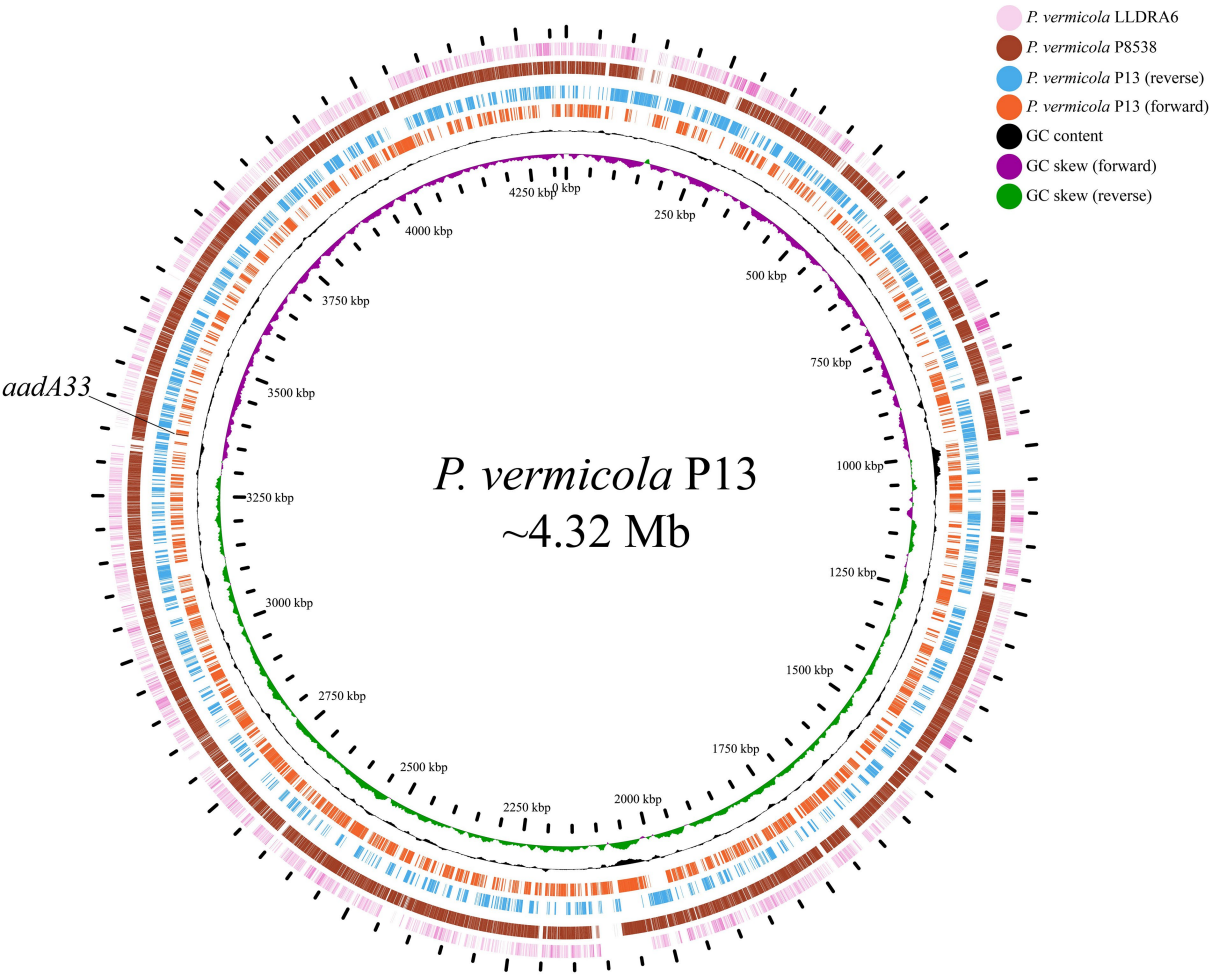
Genome maps of *Providencia vermicola* P13 and its close relatives. Circles from inside to outside represent GC skew, GC content, genes encoded in the forward and the reverse strands of the chromosome of *P. vermicola* P13, the *P. vermicola* P8538 chromosome (CP048796.1), and the *P. vermicola* LLDRA6 chromosome (CP067099.1), respectively. Regions with <80% nucleotide identities with that of the *P. vermicola* P13 chromosome are left blank.

The whole genome of *P. vermicola* P13 consists of one chromosome (plasmid-free), approximately 4.32 Mb in length, with 41.0% GC content, and encodes 3,919 open reading frames (Table 4). Comparative genomic analysis revealed that the genomes of *P. vermicola* P8538 (93.0% coverage and 99.33% identity) and *P. vermicola* LLDRA6 (78.0% coverage and 90.20% identity) showed the highest similarities with that of *P. vermicola* P13 (Figure 1).

**Table 4:** General features of the *P. vermicola* P13 genome.

Description	Chromosome
Size (bp)	4,324,465
GC content (%)	41.0
Predicted coding sequences (CDSs)	3,819
Known proteins	2,674
Hypothetical proteins	1,145
Protein coding (%)	97.45
Average ORF length (bp)	933
Average protein length (aa)	315
tRNAs	77
rRNA operons	(16S-23S-5S) × 22

### Phenotypic and genotypic characterization of antibiotic resistance of *Providencia vermicola* P13

The *in vitro* susceptibility test showed that *P. vermicola* P13 exhibited resistance to many tested antimicrobials, including aminoglycosides (such as spectinomycin, streptomycin, tobramycin, gentamicin, and kanamycin), β-lactams (ampicillin, cefoxitin, ceftazidime, and meropenem), quinolones (levofloxacin), chloramphenicol, tetracycline, fosfomycin, and polymyxin E. A total of 18 genes (15 genotypes) with ≥95% similarity to the antibiotic resistance genes in the CARD database were identified on the chromosome, including four genotypes of aminoglycoside modifying enzymes (*aadA2*, *aph(3′)-Ia*, *aph(4)-Ia* and *aac(3)-IV*) and two genotypes of β-lactamase (*bla*_NDM-1_ and *bla*_OXA-10_) (Table 5). It should be noticed that although the strain showed resistance to spectinomycin (MIC >2048 μg/ml) and streptomycin (MIC 256 μg/ml) (Table 2), no function-characterized gene which conferred resistance to spectinomycin or/and streptomycin was identified. There might be a novel mechanism responsible for the resistance phenotype against the two antimicrobials of the bacterium. Spectinomycin and streptomycin were usually substrates of *aadA* (Ramirez and Tolmasky, 2010). When analyzing the annotation result of the genome, we found that one predicted hypothetical gene encoding a protein (finally designated *aadA33*) which shares 91.57% coverage and 49.37% amino acid identity of AadA5 (AAF17880.1) in the CARD database, a protein that mediated resistance to spectinomycin and streptomycin (Sandvang, 1999), was found. To figure out whether this hypothetical gene was related to the phenotype of the bacterium resistant to spectinomycin and streptomycin, the gene was cloned, and its function was determined.

**Table 5:** Resistance genes identified in *P. vermicola* P13.

Bacterium	P13
Resistance genes	Chromosome
Aminoglycoside modifying enzyme	*aac(3)-IV*
	*aadA2*
	*aph(3′)-Ia*
	*aph(4)-Ia*
ABC-F ATP-binding cassette ribosomal protection protein	*msrE*
Chloramphenicol acetyltransferase	*catB8*
Lincosamide nucleotidyltransferase	*linG*
Macrolide phosphotransferase	*mphE*
Major facilitator superfamily antibiotic efflux pump	*qacE*Δ*1*
	*floR*
β-Lactamase	*bla*_NDM-1_
	*bla*_OXA-10_
Sulfonamide resistant	*sul1*
	*sul2*
Trimethoprim resistant dihydrofolate reductase	*dfrA1*

### *aadA33* confers resistance to spectinomycin and streptomycin

Compared with the control strain (pUCP20/DH5α), the recombinant carrying *aadA33* (pUCP20-*aadA33*/*E. coli* DH5α) exhibited >256- and 128-fold increase in MIC levels of spectinomycin and streptomycin, respectively. However, no significant increase in the MIC level was identified for the other tested aminoglycosides (Table 2). The enzyme can catalyze adenylylation of spectinomycin and streptomycin with *k*_cat_/*K*_m_ of 3.28 × 10^4^ (M^−1^ s^−1^) and 3.37 × 10^4^ (M^−1^ s^−1^), respectively (Table 6). The kinetic parameters displayed that the substrates of AadA33 are consistent with its MIC patterns.

**Table 6:** Kinetic parameters of AadA33.

Substrate	*k*_cat_ (s^−1^)	*K*_m_ (M)	*k*_cat_/*K*_m_ (M^−1^ s^−1^)
Spectinomycin	6.03 × 10^−1^	1.84 × 10^−5^	3.28 × 10^4^
Streptomycin	5.36 × 10^−1^	1.59 × 10^−5^	3.37 × 10^4^
Tobramycin	NA^a^	NA^a^	NA^a^

a NA, no hydrolysis detected.

### Comparative analysis of the *aadA33* gene and its relatives

**Figure 2:**
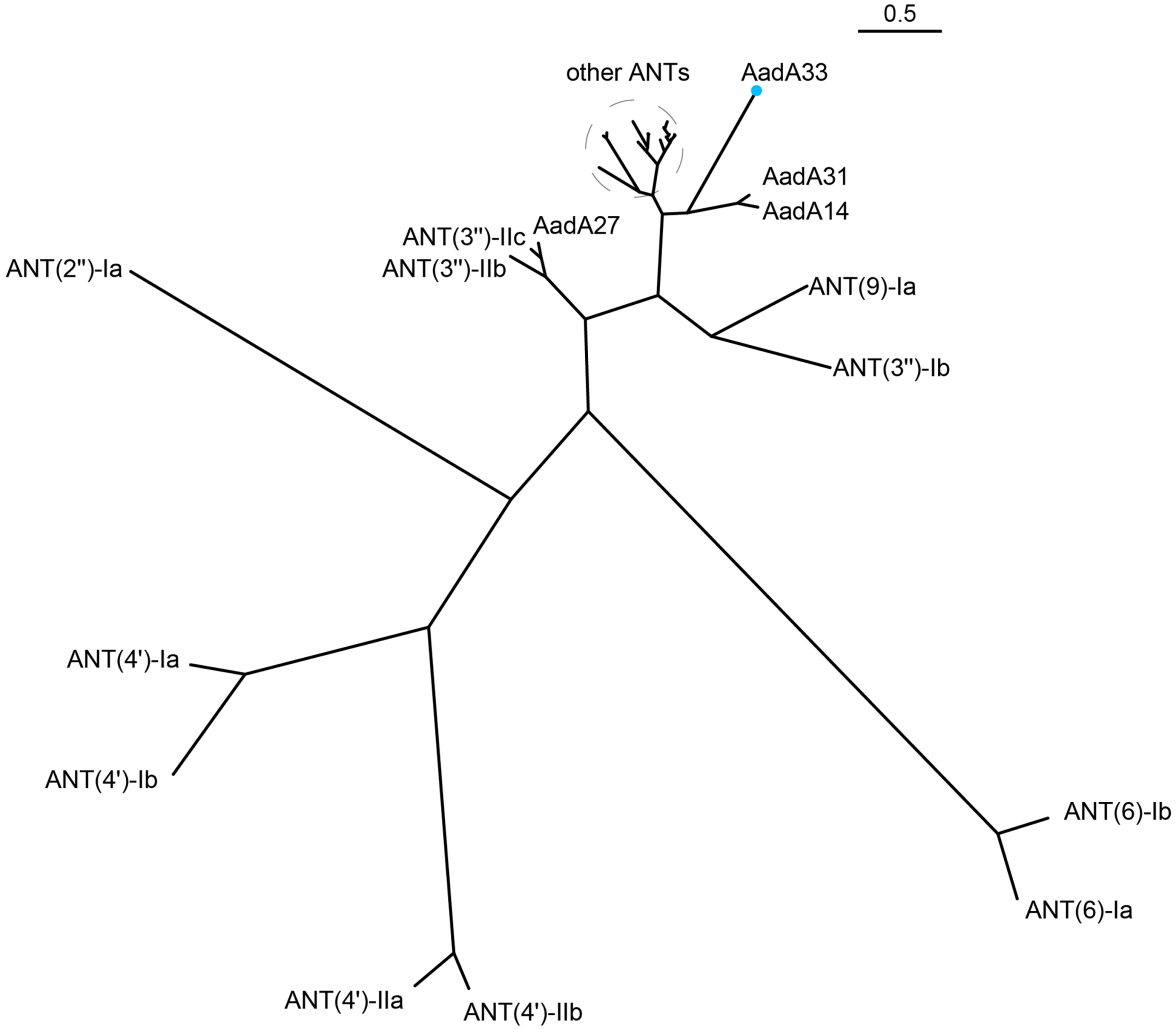
A phylogenetic tree showing the relationship of AadA33 with other functionally characterized ANTs. AadA33 is highlighted with a blue dot. Other ANTs include: AadA1, AadA4, AadA5, AadA6, AadA7, AadA8, AadA9, AadA10, AadA11, AadA13, AadA17, AadA21, AadA23, AadA24, AadA25, AadA28, AadA29, AadA30, ANT(3″)-IIa.

**Figure 3:**
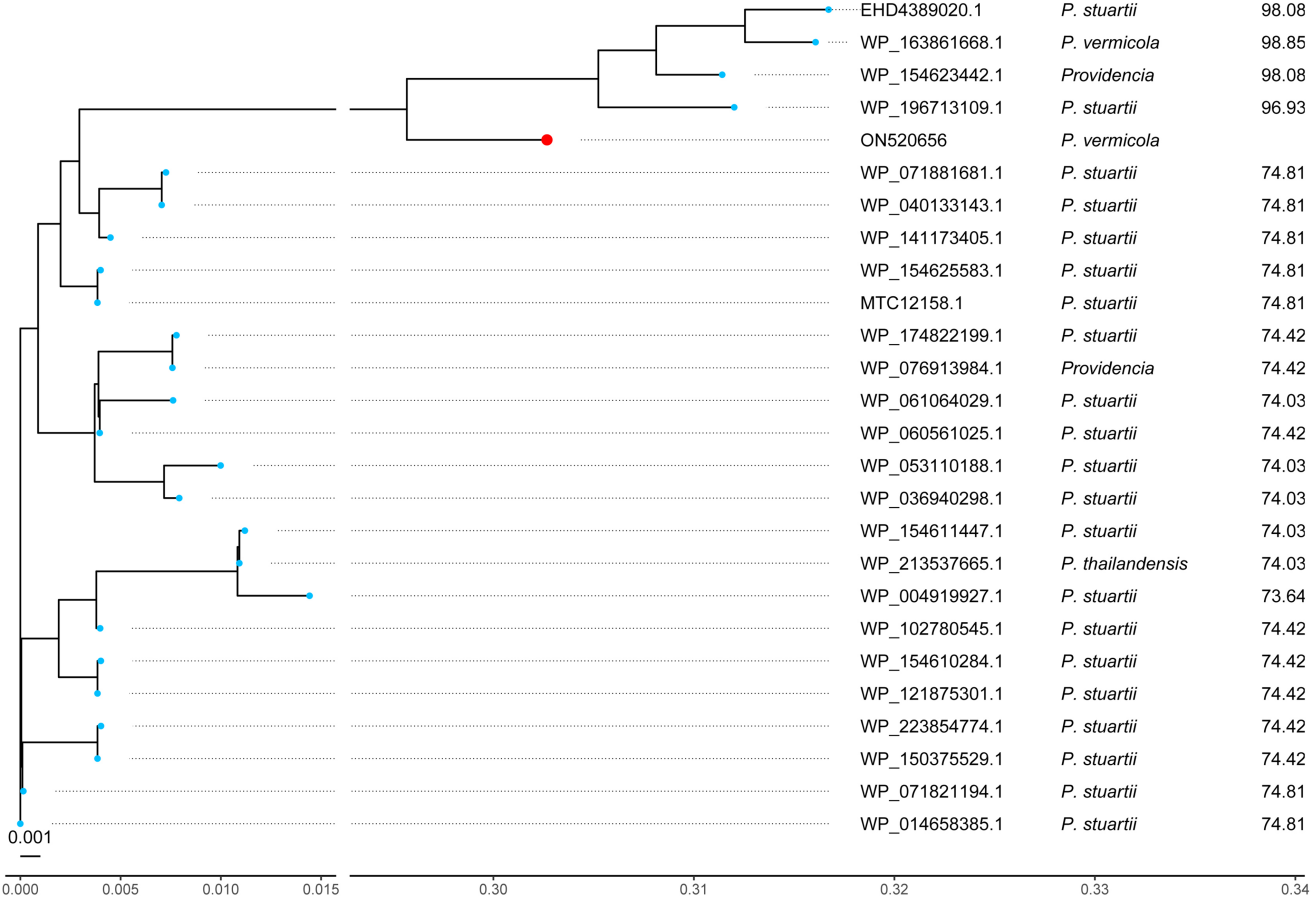
A phylogenetic tree showing the relationship of AadA33 with other putative ANTs. AadA33 is highlighted with a red dot. The three columns on the right represent accession numbers, the taxonomy of that sequence, and amino acid identity (%) with AadA33, respectively.

**Figure 4:**
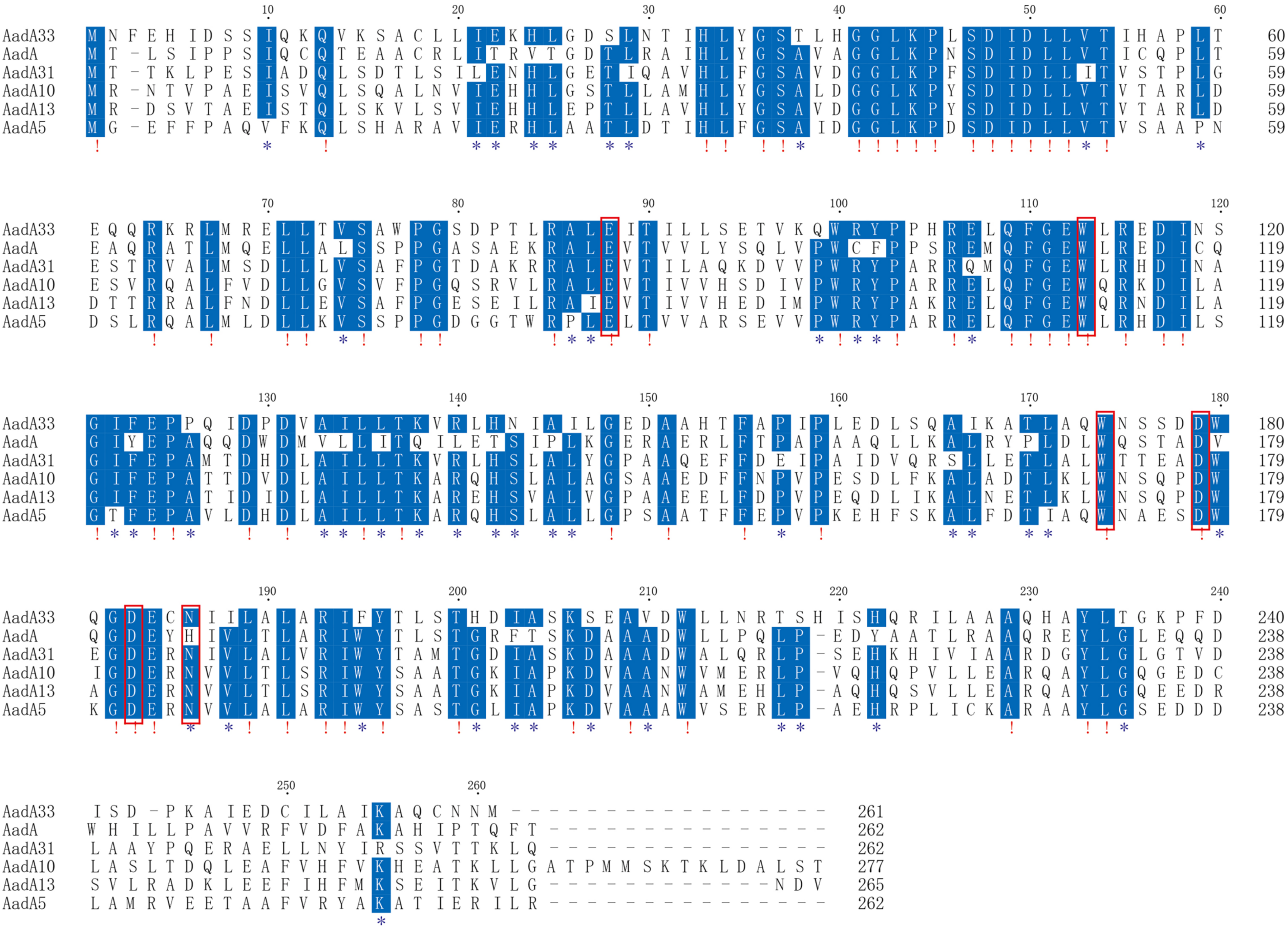
Multiple sequence alignment of AadA33 with other close relatives. Exclamations indicate fully conserved residues; asterisks indicate strongly similar residues; gaps are represented using hyphens. The numbers on the right represent the corresponding sequence length. The red frames indicate functional residues. Accession numbers of AadA proteins: AadA (Q8ZPX9), AadA31 (AUX81654.1), AadA10 (AAL36430.1), AadA13 (ABW91178.1) and AadA5 (AAF17880.1).

Phylogenetic analysis of the AadA33 with ANT(3″)-Ia family and other function characterized ANTs revealed that AadA33 has a close relationship with AadA14 and AadA31. It suggests that AadA33 is a novel lineage of the ANT(3″)-Ia family (Figure 2). Located on the chromosome, the *aadA33* gene is 786 bp in length and encodes a 261 aa protein with a molecular weight of 29.3 kDa (Supplementary Figure S1) and a p*I* value of 5.59. When searching the homologous sequences of *aadA33* in the NCBI non-redundant nucleotide and protein database. 25 protein sequences with >70% identity were found and they were all from the genus *Providencia* (Figure 3). The deduced protein sequence of AadA*33* shared the highest amino acid similarity (100% coverage and 98.85% identity) with the DUF4111 domain-containing protein (WP_163861668.1) encoded on the chromosome of *P. vermicola* P8538 (NZ_CP048796). The other 24 sequences with similarities ranging from 73.64% to 98.08% were from *P. stuartii* (83.3%, 20/24), unclassified *Providencia* (12.5%, 3/24), and *P. thailandensis* (4.2%, 1/24). Besides, protein sequences with >90% identity against AadA33 were also found in 4 out of 25 whole-genome sequenced clinical *Providencia* isolates (data not shown). However, AadA33 only shared highest identity of 51.28%, 51.09%, 49.37%, 47.22% and 45.77% amino acid identities with the function characterized AadA31 (AUX81654.1), AadA (Q8ZPX9), AadA5 (AAF17880.1), AadA10 (AAL36430.1) and AadA13 (ABW91178.1), respectively. To analyze the resistance function-related structural mechanism of AadA33, multiple sequence alignment of AadA33 and the function-characterized AadA proteins including the structure-characterized AadA (Q8ZPX9) was built (Figure 4). It turns out that AadA33 contains four amino acid residues (E88, W113, D183, and N186) responsible for the adenylation of spectinomycin, and two residues (W174 and D179) for streptomycin (Stern et al., 2018).

**Figure 5:**
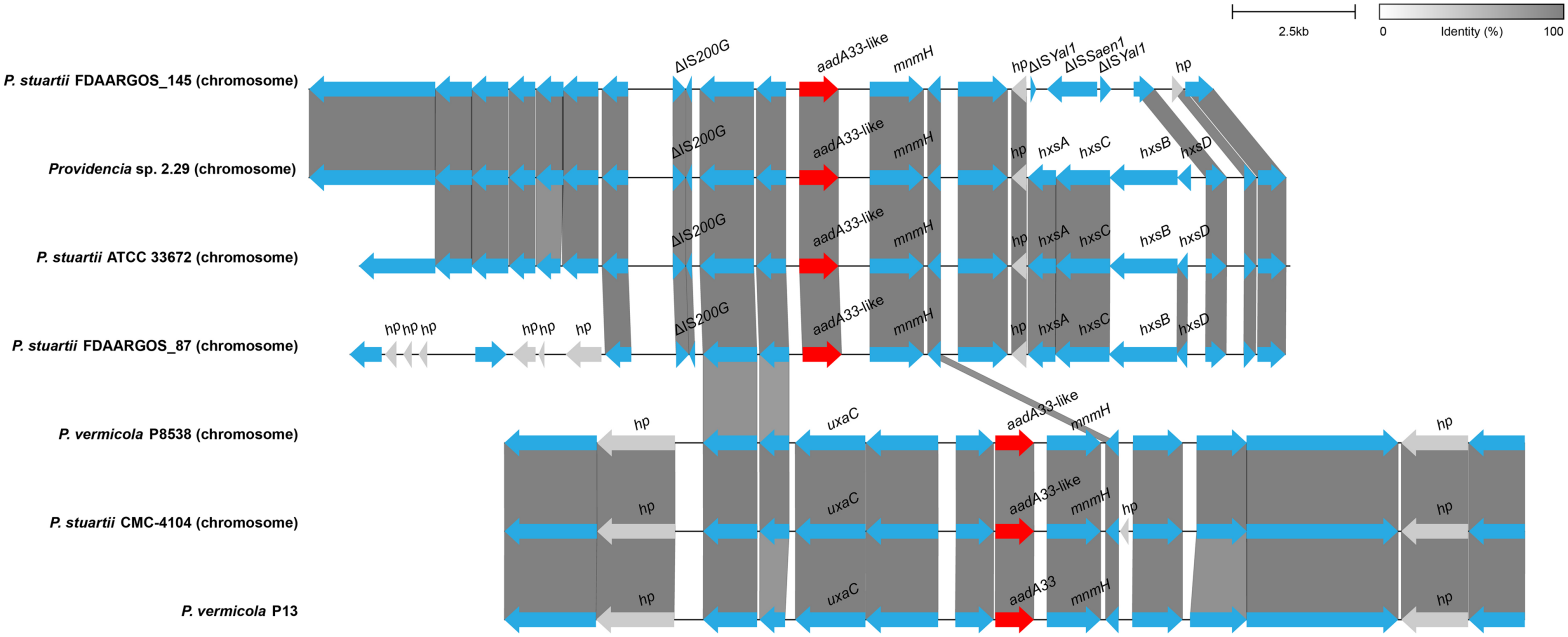
Genetic environment of the *aadA33* and *aadA33*-like genes. Regions with ≥80% amino acid identity were colored grey. Accession numbers: *Providencia stuartii* FDAARGOS_145 (NZ_CP014024.2), *Providencia* sp. 2.29 (NZ_CP065420.1), *P. stuartii* ATCC 33672 (NZ_CP008920.1), *P. stuartii* FDAARGOS_87 (NZ_CP031508.1), *P. vermicola* P8538 (NZ_CP048796.1) and *P. stuartii* CMC-4104 (CP095443.1). *hp*: hypothetical protein.

To figure out the genetic context of *aadA33*, the sequences of about 20 kb in length with an *aadA33*-like gene (with >70 identity to *aadA33*) at the center were retrieved from the NCBI non-redundant nucleotide database (Figure 5). No mobile genetic element was found in the adjacent regions of *aadA33*. Among the 16 fragments containing the *aadA33*-like sequences, most (87.5%, 14/16) were from *P. stuartii*. The *aadA33* encoding fragment of *P. vermicola* P13 is particularly similar (100% coverage and 99.31% identity) to that of *P. vermicola* P8538 and *P. stuartii* CMC-4104.

## Discussion

The *aadA33* gene is intrinsic in the genus *Providencia*. The 25 predicted (hypothetical) homologous ANT protein sequences of AadA33 in NCBI non-redundant protein database and 4 homologous sequences in clinical strains are all from genus *Providencia*. While some other *aadA* genes such as *aadA5* (Sandvang, 1999), *aadA13* (Revilla et al., 2008), and *aadA14* (Kehrenberg et al., 2005) were found related to mobile genetic elements (MGEs) encoded on plasmids or the chromosomes, *aadA33* was not associated with the MGEs, which suggested that it might be intrinsic in this strain.

AadA belongs to the ANT(3″) gene family. The resistance profile of AadA33 is consistent with the members in the ANT(3″)-Ia group. Like the other AadA enzymes such as AadA10 (Partridge et al., 2002), AadA13 (Revilla et al., 2008), and AadA14 (Kehrenberg et al., 2005), it confers resistance to streptomycin and spectinomycin. Besides, four aminoglycoside 3″-nucleotidyltransferases in the ANT(3″) family with the identities from 33.04% to 47.62% with AadA33, including ANT(3″)-IIa (CAA26199.1), ANT(3″)-IIb (ENU91137.1), ANT(3″)-Ib (QEQ43477.1) and ANT(3″)-IIc (ENU37733.1) were also confirmed to be resistant to streptomycin and spectinomycin (Zhang et al., 2017; Ruiz et al., 2019). It has been shown that ANTs of the different groups showed different resistance spectra. ANT(6)-Ia and ANT(6)-Ib mediate resistance to streptomycin, while ANT(9)-Ia, however, showed resistance to spectinomycin. ANT(2″) confers resistance to gentamicin, dibekacin, kanamycin, sisomicin, and tobramycin, while ANT(4′) mediates resistance to amikacin, dibekacin, isepamicin, and tobramycin. Although AACs and APHs mediate modification of a wide range of aminoglycosides including amikacin, gentamicin, dibekacin, kanamycin, tobramycin, and neomycin, only APH(6)-Ic, APH(6)-Id and APH(3″)-Ic confer resistance to streptomycin, and APH(9) to confer resistance to spectinomycin.

AadA33 contains one conserved protein domain family PRK13746 (aminoglycoside resistance protein). It has been validated that the determinants of spectinomycin and streptomycin resistance of AadA (Q8ZPX9) conferring adenylation on spectinomycin were E87, W112, D182, and H/N185, and on streptomycin are W173 and D178 (Stern et al., 2018). 86E, 180D, and 183N in ANT(9) is essential for its spectinomycin resistance (Kanchugal and Selmer, 2020). Although AadA33 shares an overall low identity of 51.09% with this AadA protein sequence (Q8ZPX9), the six amino acid residues are conserved in AadA33 (with N186 in AadA33). This further confirms the novel resistance gene of this work to be a member of the AadA group.

## Conclusion

In this work, we reported a novel aminoglycoside modifying enzyme named AadA33 from the chromosome of *P. vermicola* P13 isolated from a patient. Encoded in the chromosome, *aadA33* was not related to a mobile genetic element. It belongs to the ANTs family and shares the highest amino acid identity with an aminoglycoside *O*-nucleotidyltransferase AadA31. The novel aminoglycoside modifying enzyme confers strong resistance to streptomycin and spectinomycin, which will be beneficial for the study of the intrinsic resistance mechanism against aminoglycosides in opportunistic pathogens.

## Data availability statement

The datasets presented in this study can be found in online repositories. The names of the repository/repositories and accession number(s) can be found in the article/Supplementary material.

## Ethics statement

Individual patient data was not involved, and only anonymous clinical residual samples during routine hospital laboratory procedures were used in this study. It was approved by the ethics committee of the Second Affiliated Hospital and Yuying Children’s Hospital of Wenzhou Medical University, Wenzhou, Zhejiang, China.

## Author contributions

KL, HZ, YH, QB, and XL: conceived and designed the experiments. CF, MG, WJ, WS, AL, SL, LZ, and JL: performed the experiments. CF, MG, XZ, QL, HL, QB, and XL: data analysis and interpretation. CF, MG, QB, and XL: drafting of the manuscript. All authors contributed to the article and approved the submitted version.

## Funding

This study was supported by the Science & Technology Project of Wenzhou City, China (N20210001 and Y2020112), Zhejiang Provincial Natural Science Foundation of China (LY19C060002 and LQ17H190001), and the Natural Science Foundation of China (81973382).

## Conflict of interest

The authors declare that the research was conducted in the absence of any commercial or financial relationships that could be construed as a potential conflict of interest.

## Publisher’s note

All claims expressed in this article are solely those of the authors and do not necessarily represent those of their affiliated organizations, or those of the publisher, the editors and the reviewers. Any product that may be evaluated in this article, or claim that may be made by its manufacturer, is not guaranteed or endorsed by the publisher.

## Footnotes

^1^ https://www.cdc.gov/narms/antibiotics-tested.html

^2^ https://github.com/arpcard/rgi

^3^ https://www.ncbi.nlm.nih.gov/Structure/cdd/wrpsb.cgi

^4^ https://web.expasy.org/protparam/

^5^ https://ftp.ncbi.nlm.nih.gov/entrez/entrezdirect/

## Supplementary material

SUPPLEMENTARY FIGURE S1 SDS-PAGE of AadA33. Lane 1: PageRuler Prestained Protein adder (Thermo Fisher Scientific, product code: 26616); lane 2 and 3: uncleaved AadA33 with His6 tag; lane 4: cleaved AadA33 with thrombin.
