# Supplementary figures and images for: Identification of a novel aminoglycoside O-nucleotidyltransferase AadA33 in Providencia vermicola
Source: Front Microbiol. 2022 Sep 13;13:990739. doi: 10.3389/fmicb.2022.990739 (PMC9513248; doi:10.3389/fmicb.2022.990739)

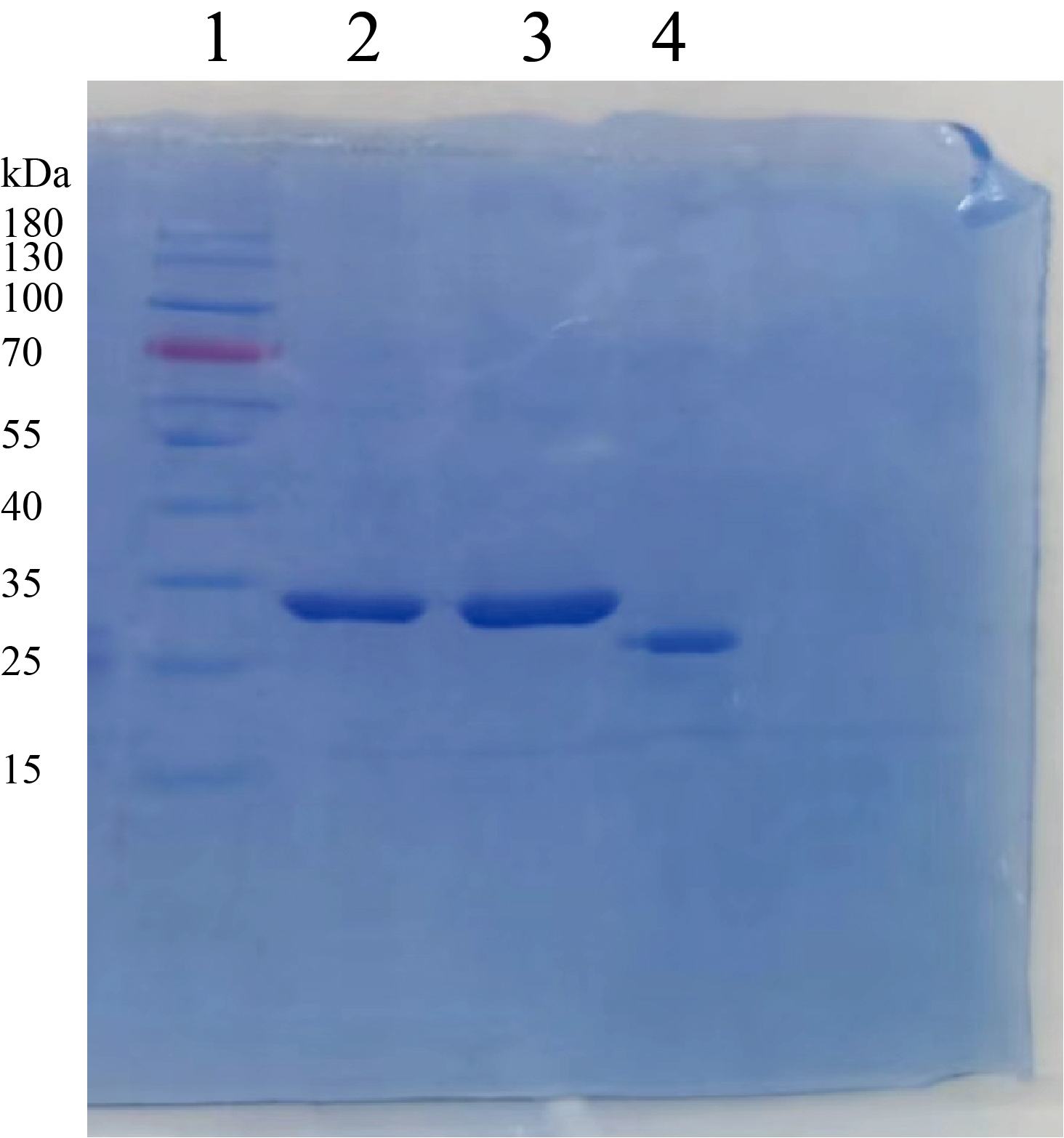

Supplement: SUPPLEMENTARY FIGURE S1 — SDS-PAGE of AadA33. Lane 1: PageRuler Prestained Protein adder (Thermo Fisher Scientific, product code: 26616); lane 2 and 3: uncleaved AadA33 with His6 tag; lane 4: cleaved AadA33 with thrombin. [file Image_1.JPEG]
